# Minimally Invasive Surgical Treatment of Acute Epidural Hematoma: Case Series

**DOI:** 10.1155/2016/6507350

**Published:** 2016-04-06

**Authors:** Weijun Wang

**Affiliations:** Department of Neurosurgery, Qiannan People's Hospital, Qiannan 558000, China

## Abstract

*Background and Objective*. Although minimally invasive surgical treatment of acute epidural hematoma attracts increasing attention, no generalized indications for the surgery have been adopted. This study aimed to evaluate the effects of minimally invasive surgery in acute epidural hematoma with various hematoma volumes.* Methods*. Minimally invasive puncture and aspiration surgery were performed in 59 cases of acute epidural hematoma with various hematoma volumes (13–145 mL); postoperative follow-up was 3 months. Clinical data, including surgical trauma, surgery time, complications, and outcome of hematoma drainage, recovery, and Barthel index scores, were assessed, as well as treatment outcome.* Results*. Surgical trauma was minimal and surgery time was short (10–20 minutes); no anesthesia accidents or surgical complications occurred. Two patients died. Drainage was completed within 7 days in the remaining 57 cases. Barthel index scores of ADL were ≤40 (*n* = 1), 41–60 (*n* = 1), and >60 (*n* = 55); scores of 100 were obtained in 48 cases, with no dysfunctions.* Conclusion*. Satisfactory results can be achieved with minimally invasive surgery in treating acute epidural hematoma with hematoma volumes ranging from 13 to 145 mL. For patients with hematoma volume >50 mL and even cerebral herniation, flexible application of minimally invasive surgery would help improve treatment efficacy.

## 1. Introduction

Acute epidural hematoma is one of the most common secondary brain neurosurgical skull injuries, accounting for 30% of intracranial hematomas. Bleeding is mainly due to ruptured meningeal middle artery [1] and partially to bleeding of dural arteriovenous fistulas and the baffle plate. Most patients mainly present with consciousness dysfunction to some extent, headache, and vomiting [2]. If simple epidural hematomas are treated immediately, a good prognosis is often achieved. However, if hematomas are accompanied with severe contusion and laceration brain injury or cerebral herniation, prognosis is usually uncertain, and death rate could reach 20% [3]. Currently, surgical indications for clearance of epidural hematoma by craniotomy are as follows [4]: (1) hematoma >40 mL and located at the supratentorial region or hematoma >10 mL and located at the infratentorial region; (2) obvious midline structure shift (>1 cm) and ventricle or cisterna pressure; (3) intracranial pressure >2.7 kPa (270 mm H20) and progressively increasing; (4) consciousness dysfunction gradually worsening, even if bleeding amount does not meet the surgical indication criteria. Traditionally, as long as patients have one of the abovementioned indications, hematoma clearance by craniotomy is performed. The surgical outcome is acceptable. However, surgical trauma is relatively important, and general anesthesia carries inherent risks; in addition, surgery cost is relatively high. Craniotomy has negative psychological effects on patients such as fear, anxiety, and depression [5–7]. Most of the patients lacked confidence to enter into marital relationships or perform jobs and social activities due to perceived disability following craniotomy. Application of minimally invasive surgery attracts increasing attention from neurosurgeons and has been applied for the treatment of acute epidural hematoma [8–10]. Because minimally invasive surgery cannot completely achieve one-off hematoma evacuation in short time and does stop bleeding under direct visualization, it has been proposed that hematomas >30–50 mL are unsuitable for this operation [9–11]. Moreover, at present, no generalized standard indications have been adopted for this surgery, which is still in its exploratory stage. Interestingly, most minimally invasive surgeries for acute epidural hematoma were conducted in hematomas ranging from 20 to 50 mL, with good outcomes [8–12]. Indeed, detailed reports assessing minimally invasive surgical treatment for cases with larger bleeding amounts are scarce. Therefore, the present study aimed to assess minimally invasive surgery for cases with hematomas >50 mL and even cerebral herniation, exploring its application value in treatment efficiency and surgical trauma reduction.

## 2. Materials and Methods

### 2.1. Patients

59 patients with acute epidural hematoma were recruited from Department of Neurosurgery, Qiannan People's Hospital, Guizhou (China), from November 2011 to March 2015. This study was approved by the Hospital's Ethics Committee; informed consent was obtained from patients and their families; minimally invasive surgery was used as surgical treatment. Inclusion criteria were as follows: diagnosis of acute epidural hematoma confirmed by 64-slice head CT upon admission, no surgery contraindications on examination prior to surgery, and agreement by patients and their families to receive minimally invasive surgery.

### 2.2. Selection of Surgical Approaches

Patients with no overt intracranial hypertension, cerebral herniation, or surgical contraindications underwent minimally invasive surgery alone; in those with multiple epidural hematomas, drainage tube numbers were decided based on hematoma amounts (Figures 4(a)–4(c)). Combination of rapid minimally invasive surgery and clearance of hematoma by craniotomy was selected in case of cerebral herniation.

### 2.3. Timing of the Surgery

For patients with clear consciousness and not very high cranial hypertension, surgery was performed as soon as possible, within 6 h of onset; in case of consciousness dysfunction, severe cranial hypertension, or cerebral herniation, immediate preoperation preparation should be made, and bed-site hematoma aspiration by minimally invasive aspiration and drilling skull drainage were performed.

### 2.4. Follow-Up and Evaluation Parameters

Patients were followed up once monthly by telephone and return visits to the hospital, until June 2015. Head CT was conducted at the first return visit. The Barthel index score of daily activity living (ADL) was evaluated three months after surgery, and the scoring criteria were interpreted as follows: poor: severe dysfunction, score ≤40; moderate: moderate dysfunction, score of 41–60; fine: mild dysfunction, score >60. Full score was 100, when patients can fully take care of themselves. Other parameters assessed included surgical time, bleeding amount during surgery, adverse effects, hospitalization duration, surgery safety, and success rate. The criteria of success rate for minimally invasive surgery included remission of high intracranial pressure, clinical symptoms, and complete hematoma drainage.

## 3. Results

### 3.1. Subjects

A total of 59 patients, including 52 males and 7 females, were included. They ranged from 4 to 68 years old, with a median age of 32 years. Causes of hematomas included fall from height (*n* = 15), car accident (*n* = 35), and fighting (*n* = 9). Hematomas with volumes ranging from 13 to 19 mL occurred in 34 cases; 25 cases had volumes of 50–145 mL; median volume was 45 mL. Four cases had simple acute epidural hematomas and 3 presented with multiple epidural hematomas; forty-one cases had epidural hematomas accompanied with skull fracture, contusion, and brain laceration; eight cases had concomitant cerebral herniation (including one patient with diffuse axonal injury); one case also had spinal cord injury.

### 3.2. Surgical Procedures [13] and Results

**Figure 1:**
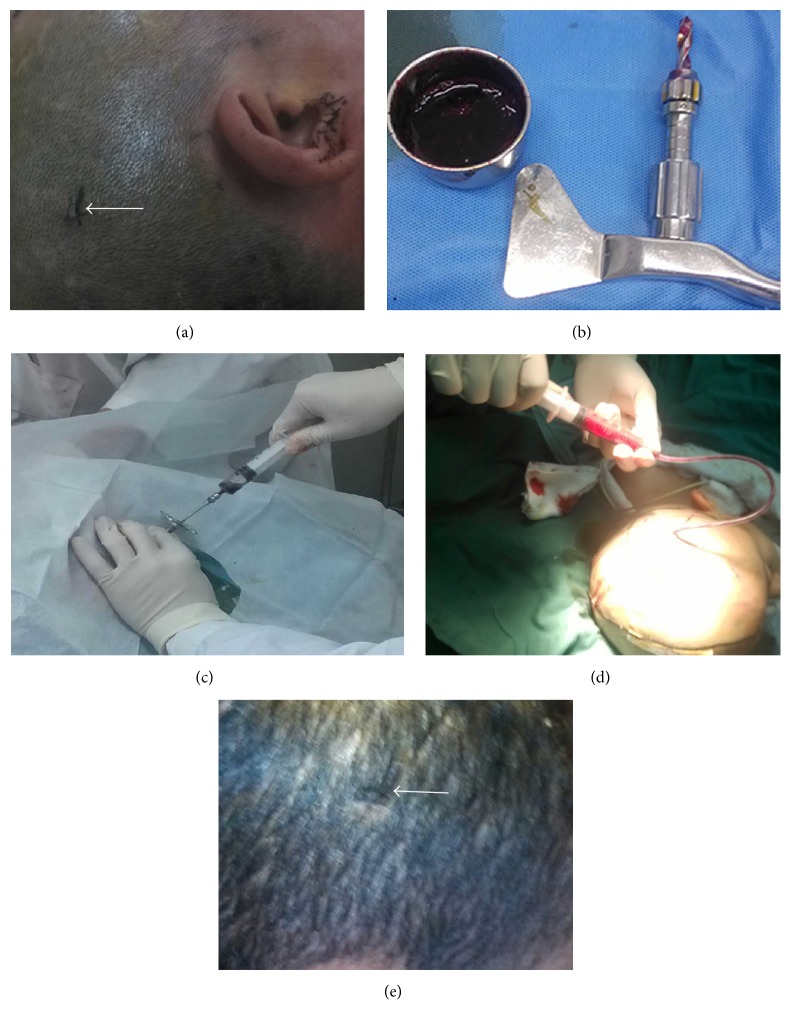
(a)–(e) Surgical procedures step by step. (a) Scalp incision with a length of 0.7 cm under local anesthesia. (b) Approximately 20 mL of blood clot was aspirated during surgery. A manual Skull driller is shown. (c) Old blood clot aspirated during surgery. (d) Drainage tube placed within the cavity of a hematoma during surgery, with old blood clot aspirated during surgery. (e) Healed incision on the scalp after surgery.

Hematoma sites and volumes were confirmed by stereotaxic head CT prior to surgery. The puncture point on the scalp was the thickest hematoma area, and puncture angle and depth were measured. Routine sterilization and draping were carried out, and 2% lidocaine was injected for local anesthesia. Once anesthesia was effective, 0.5–0.8 cm incision was made on the scalp (Figure 1(a)). A manual skull driller was used to drill through the scalp and skull (Figure 1(b)), and hematoma was aspirated as much as possible using a brain puncture needle (Figure 1(c)). A 10 F drainage tube was inserted into the hematoma cavity (Figure 1(d)); then, the tube core was withdrawn and the drainage tube further inserted toward the hematoma for 1 cm. After tube suture and fixation, a closed backflow prevention drainage device was connected. Surgery was completed within 10–20 minutes. 24 h after surgery, the hematoma was slowly rinsed with normal saline; once the washing solution was clear, 20,000–40,000 units of urokinase in 2-3 mL normal saline were injected into the hematoma cavity (urokinase amounts were adjusted, according to hematoma volumes); drainage was opened 2 h after tube closure, 1-2 times daily. For the 8 patients with cerebral herniation, hematoma was first partially aspirated under local anesthesia, and complete hematoma clearance by craniotomy was immediately performed in the surgery room. Whether bone flap remained depended on contusion severity, brain injury laceration, and intracranial pressure after surgery. Bone flap was kept in 2 cases, with decompression made by removing bone flaps in 5 cases. After surgery, patients received routine care such as oxygen delivery, hemostasis, and dehydration for lowering intracranial pressure and antibiotics for bacterial infection prevention. The antibiotics were administered 30 min before surgery, and prophylactic treatment was administered 24 h after surgery if the procedure lasted longer than 3 h [14] and brain cell nutrition. All 59 patients underwent successful hematoma aspiration and drainage tube placement. Intraoperative bleeding amounts were 5–10 mL. No surgical complications occurred, such as increased hematoma volume or functional impair caused by surgical trauma, anesthesia, or intracranial infection.

### 3.3. Postoperative Outcomes

Hematomas in 8 cases with epidural hematomas were completely cleared by craniotomy as shown by head CT at 1 day after surgery. No rebleeding occurred. Head CT was carried out anew to assess progression of hematoma drainage in 51 cases treated with simple minimally invasive surgery at 3, 5, and 7 days after surgery. Drainage tubes were withdrawn after hematoma clearance through drainage in 3, 17, and 31 cases at 3, 5, and 7 days, respectively, after surgery. The success rate of minimally invasive surgery was 100%. Median hospital stay was 10 days; follow-up time was 3 months. Two patients died; one of them had diffuse axonal injury and died the 3rd week from respiratory failure caused by pulmonary infection; the other had cerebral hernia and died from central respiratory and circulatory failure. Barthel index scores of ADL were ≤40 (*n* = 1), 41–60 (*n* = 1), and >60 (*n* = 55); scores of 100 were obtained in 48 cases, with no dysfunctions.

## 4. Discussion

The concept of minimally invasive surgery in neurosurgery is widely accepted; therefore, treatment approaches of acute epidural hematoma gradually develop from craniotomy to minimally invasive surgery [15]; indeed, reduced surgical trauma is obtained with minimally invasive surgery; low risk and rapid recovery alongside reduced cost are the other advantages. The patients understood that the procedure was associated with less neurological dysfunction, which reduced any negative physiological consequences. Multiple studies have assessed minimally invasive surgery for the treatment of acute epidural hematomas ranging from 20 to 50 mL [8–12], with good outcomes achieved. In the present study, hematoma drainage with simple minimally invasive awl cranium drainage was performed in 34 of 59 cases, with volumes ranging from 13 to 49 mL, including 2 patients with acute epidural hematoma in the infratentorial region (Figures 3(a)–3(c)); drainage became clear at 3 to 7 days after surgery, and the drainage tube was then pulled off. Barthel index scores of ADL in these patients were 100 with complete recovery and without dysfunction, corroborating previous reports [8–12].

**Figure 2:**
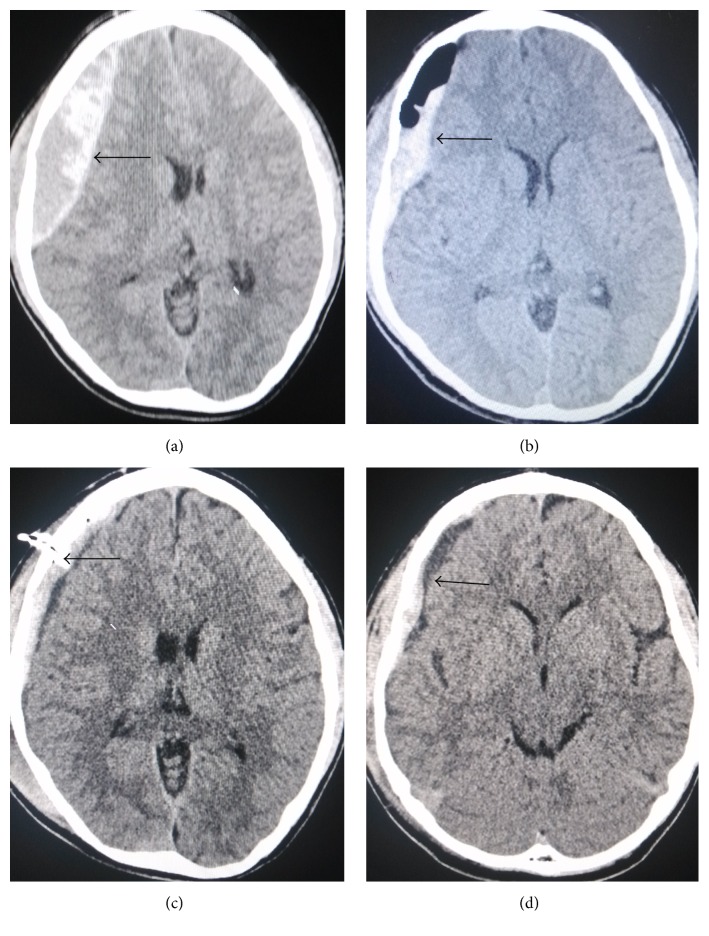
(a)–(d) Head CT images showing minimally invasive drainage treatment of acute epidural hematoma at the right temporal-parietal region, prior to and after surgery. (a) Head CT scan image prior to surgery. Hematoma volume was approximately 80 mL, midline shift. (b) Head CT scan image immediately after surgery; hematoma volume was markedly reduced with a residual amount of approximately 30 mL. (c) At 3 days after surgery, most of the hematoma has been drained; arrow indicates a drainage tube. (d) Five days after surgery, hematoma was almost cleared, and the drainage tube was pulled off.

**Figure 3:**
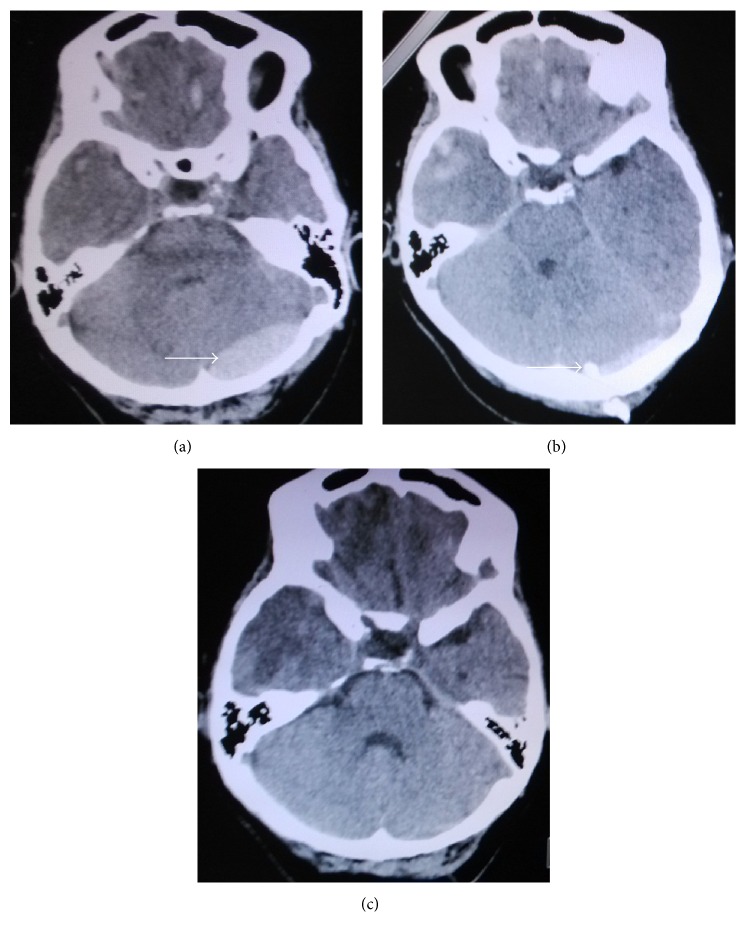
(a)–(c) Head CT images showing acute epidural hematoma at the left occipital region prior to and after surgery. (a) Head CT images prior to surgery; bleeding volume was approximately 13 mL. (b) Head CT images 3 days after surgery; hematoma volume was obviously reduced. Arrows indicate the site of hematoma and drainage tube. (c) Head CT images 5 days after surgery; hematoma was almost cleared, and the drainage tube was pulled off.

**Figure 4:**
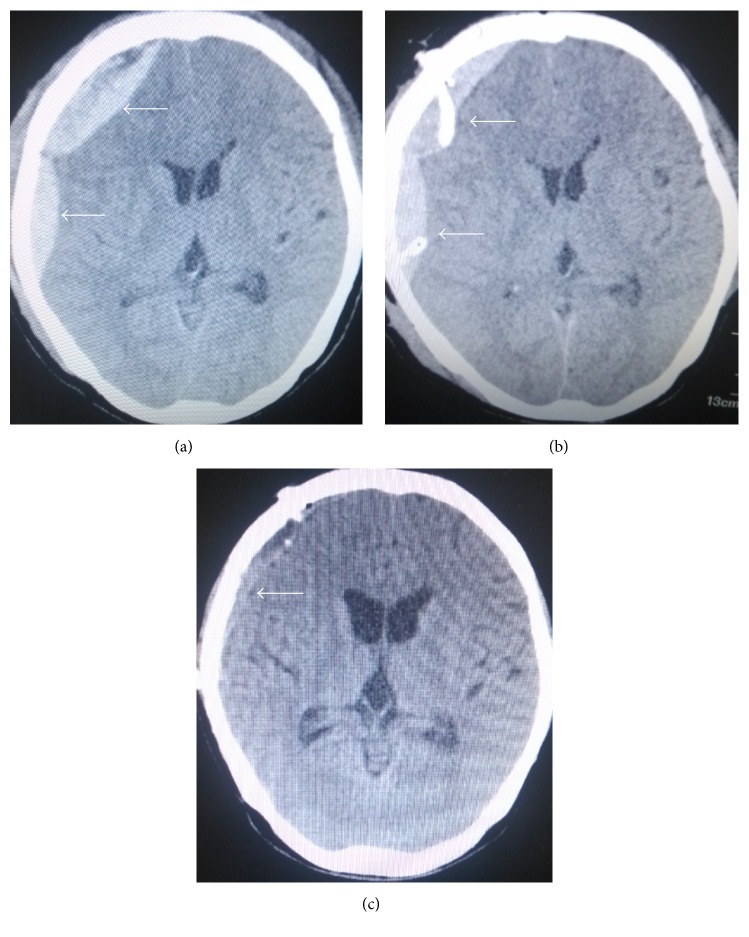
(a)–(c) Head CT images showing minimally invasive drainage treatment of acute multiple epidural hematoma at the right temporal-parietal region prior to and after surgery. (a) Head CT scan image prior to surgery. (b) Head CT scan reexamined immediately after surgery; hematoma volume at the parietal region was markedly reduced. Arrow indicates hematoma and drainage tube. (c) Five days after surgery, hematoma was almost cleared and the drainage tube was pulled off.

It was suggested that simple minimally invasive surgery should not be conducted for hematomas of more than 50 mL [6, 8, 10]. In this study, among the 25 cases with hematomas ranging from 50 to 145 mL, 17 underwent simple minimally invasive surgery (with the largest hematoma up to 80 mL, Figure 2(a)); the hematomas were completely drained within 7 days, and drainage tubes were pulled off. Barthel index scores were 100 in 12 cases at discharge, and 3 cases had scores >60. Although hematoma volumes were >50 mL in these patients, consciousness dysfunctions were not severe, and no cerebral herniation occurred; the fluid within hematomas in some cases was not sticky and could be more aspirated during surgery (Figure 2(b)). Some residues were maintained inside the hematomas; therefore, intracranial pressure was obviously relieved. Some cases with hematoma in elderly people were accompanied with brain atrophy; there is certain space with buffering capacity within the skull, and thus minimally invasive surgery can be still applied for these cases.

**Figure 5:**
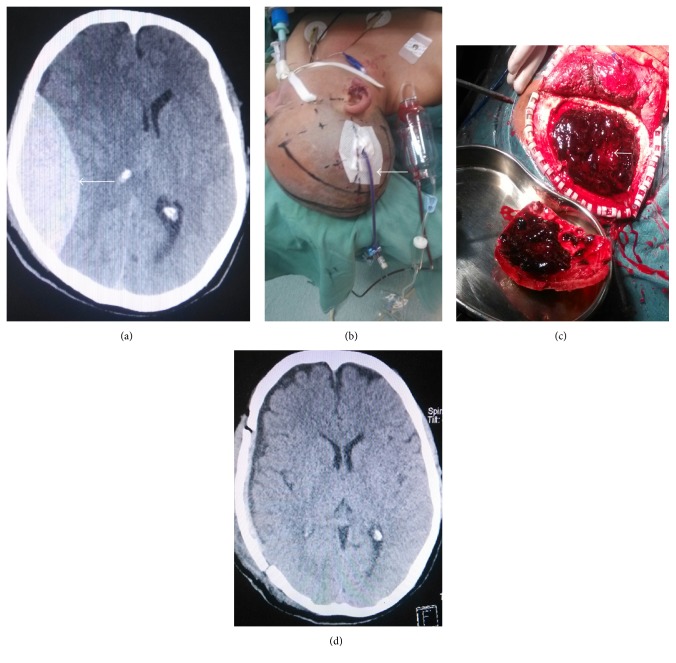
(a)–(d) Head CT images showing a huge acute epidural hematoma with cerebral hernia at the right temporal-parietal region prior to and after surgery. (a) Head CT scan image prior to surgery; hematoma volume was approximately 145 mL and midline shift 13 mm. (b) Hematoma volume with rapid minimally invasive aspiration under local anesthesia was approximately 40 mL; drainage tube was placed. (c) Skull bone flap was opened during surgery; an arrow indicates a partly aspirated hematoma in the hematoma center. (d) Head CT scan reexamined the day after surgery; drainage of hematoma was almost complete, brain tissues were expanded again, midline shift was improved, dilated pupil is recovered and light reflection was recovered.

The other 8 cases with cerebral hernia underwent minimally invasive surgery in combination with craniotomy. Two cases had long-term coma after surgery and died from complications. The Barthel index scores were <40 in one case with concomitant cervical spinal injury; scores were 40–59 in one case, >60 in two cases, and 100 in two cases. For patients with acute epidural hematoma accompanied with cerebral hernia, it took 1.5–2 h from admission, presurgery discussion, signature, presurgical preparation, general anesthesia by endotracheal intubation, incision of scalp flap, drilling of the skull, opening of bone flap, and clearance of hematoma to intracranial pressure decompression. In traditional surgical approaches for hematoma clearance by craniotomy, it takes at least 1.5–2 h to reduce cerebral hernia. According to intracranial volume-pressure relationships, patients already with cerebral hernia showed relatively high intracranial pressure; even hematoma was reduced by a small volume, likely leading to overtly reduced intracranial pressure. Based on this mechanism, minimally invasive surgery cannot completely clear hematomas at once and does not yield fully decompression; nevertheless, it can help partly aspirate hematomas within short time to partially reduce high intracranial pressure. Moreover, prerequisite for minimally invasive surgery is not high, and the surgery can be performed under local anesthesia before preoperative preparation for craniotomy under general anesthesia. In addition, surgery time is only 10–20 minutes. In the present study, there were 8 hematoma cases accompanied with cerebral hernia, with the largest hematoma volume of 145 mL (Figure 5(a)); aspiration volume was 30–40 mL with minimally invasive surgery, and patients were immediately sent to surgery room for routine clearance of hematoma by craniotomy. As shown in Figure 5(c), a part of aspirated hematoma was seen in the hematoma center; bone flap can be maintained if intracranial pressure is not high during surgery (Figure 5(d)) [16]. During surgical treatment of patients with cerebral herniation, minimally invasive surgery can help partly aspirate hematomas during preoperative preparation for craniotomy, rapidly decompressing hematoma partially, saving time, and providing conditions for recovery.

Although this surgical approach is simple, cautions should be observed during operation; complications should be avoided as much as possible. The thickest part of hematoma was chosen as the puncture site. Most epidural hematomas are accompanied with skull fracture. If the thickest hematoma region is a fracture line, it should be avoided as much as possible to prevent aggravation of fracture injury by skull drilling, as well as the possibility of bleeding. If patients had comminuted fracture of the skull, the fracture site should be avoided as well; otherwise, it is possible to cause dislocation of fracture fragments or even collapse, leading to bleeding again, or brain damage. Meanwhile, the pterional should be avoided in order to prevent damage of the meningeal middle artery, which also causes bleeding. During surgery, after successful skull drilling, if hematoma is a thin liquid, a drainage tube will be directly inserted for aspiration; in case of a thick liquid, because the silicone drainage tube is soft, stronger aspiration might cause closure of the cavity, limiting the aspirated volume. It is better to use brain puncture needle for aspiration; during aspiration, direction is continuously changed, or the needle is rotated to aspirate as much as possible. With a large hematoma and limited aspiration volume, a drainage tube with double holes is used for aspiration, which has advantage in speeding up hematoma drainage. Before injection of urokinase into hematoma cavity after surgery, it is preferable to rinse the cavity with small hematoma amounts many times, which is beneficial in shortening drainage time; epidural space is a relative safe environment; if hematoma volume is large, frequency of urokinase injection can be increased up to 2 times daily.

In summary, the indications for single minimally invasive surgery include (1) mild impairment in consciousness, sleepiness, or lethargy; (2) cerebral hematoma volume larger than 20 mL or cerebellar hematoma volume larger than 10 mL; (3) hematoma volume larger than 50–80 mL but with limited disorder of consciousness, or lower density of hematoma, elderly patients with severe brain atrophy, or minor contusion and laceration of brain. The indications for minimally invasive surgery before emergency measures prior to craniotomy include (1) severely impaired consciousness or coma; (2) severe contusion and laceration of brain with hematoma volumes ranging between 20 mL and 50 mL; and (3) cerebral hernia with the volume of hematoma larger than 50 mL to 80 mL. With the development of minimally invasive surgery, its application spectrum gradually increases; treatment efficiency is continuously improved as well. Due to minor surgical trauma, relatively low requirement for surgery reduced anesthesia risk, simple operation procedure, and low cost; minimally invasive surgery is a popular choice among doctors and patients. However, prognosis of acute epidural hematoma is affected by multifactorial conditions. In particular, treatment effects are still worrisome for patients with large contusion and laceration brain injury, and with long time cerebral herniation; meanwhile, minimally invasive surgery cannot achieve complete clearance of hematoma within short time. If hematoma cannot be drained to a certain amount to reduce intracranial pressure at the peak of edema, cerebral herniation might occur, and patients must undergo craniotomy. Hemostasis cannot be carried out under direct visualization, and hematoma volume could increase during surgery; consequently, patients might need to undergo craniotomy. The sample size was relatively small in the present study, and larger sample size studies should be carried out. Flexible application of minimally invasive surgery based on individuals' conditions is beneficial for improving the treatment outcome of acute epidural hematoma.

## 5. Conclusions

Minimal invasive surgery has many advantages such as small incisions, quicker operative time, and low anesthesia risk. It has good curative effect on treatment of the acute epidural hematoma (AEDH) with a small amount of bleeding (less than 50 mL) and can avoid craniotomy. Besides, for AEDH with a large amount of bleeding (more than 50 mL), minimal invasive surgery can also improve the therapeutic effects and achieve effectiveness with tiny incisions.
